# Evolution of VOC and Sensory Characteristics of Stracciatella Cheese as Affected by Different Preservatives

**DOI:** 10.3390/foods9101446

**Published:** 2020-10-12

**Authors:** Giuseppe Natrella, Graziana Difonzo, Maria Calasso, Giuseppe Costantino, Francesco Caponio, Michele Faccia

**Affiliations:** Department of Soil, Plant and Food Sciences, University of Bari, Via Amendola 165/A, 70126 Bari, Italy; giuseppe.natrella@uniba.it (G.N.); graziana.difonzo@uniba.it (G.D.); maria.calasso@uniba.it (M.C.); giuseppe.costantino@uniba.it (G.C.); francesco.caponio@uniba.it (F.C.)

**Keywords:** stracciatella cheese, volatile organic compounds, sensory characteristics, natural preservatives, cheese storage

## Abstract

Undesired volatile organic compounds (VOCs) can negatively affect the flavor of fresh food products; especially those characterized by a mild and delicate aroma. Finding connections between chemical and sensory analyses is a useful way to better understand the arising of off-flavors. A study was conducted on stracciatella; a traditional Italian cream cheese that is emerging on international markets. Samples were prepared by adding two different preservatives (alone or combined): sorbic acid and an olive leaf extract. Their influence on flavor preservation during refrigerated storage was investigated by chemical, microbiological and sensory analyses. A strong change of the VOC profile was ascertained after 8 days in the control cheese and in the sample added with leaf extract alone. The samples containing sorbic acid, alone or in combination with leaf extract, gave the best chemical and sensory results, demonstrating a significant shelf-life extension. In particular, these samples had lower concentrations of undesired metabolites, such as organic acids and volatiles responsible for off-flavor, and received better scores for odor and taste. Ex and Ex-So samples had significantly higher antioxidant activity than Ctr and So throughout the entire storage period, and the color parameter shows no differences among samples taken on the same day. The use of the olive leaf extract, at the concentration tested, seemed to be interesting only in the presence of sorbic acid due to possible synergic effect that mainly acted against Enterobacteriaceae.

## 1. Introduction

One of the decay’s symptoms of an expiring food is its smell [1,2]. Thus, the study of the volatile organic compound (VOC) profile can be very useful for establishing the organoleptic status of the product. The onset of off-flavor can be caused by many factors, such as food handling, processing, spoilage microorganism and lipid oxidation [3,4,5]. Many researchers have investigated the VOC responsible of off-flavor and have tried to link them to specific sensory descriptors [5,6,7,8,9,10]. The fastest way to analyze these compounds is using SPME-GC/MS (Solid Phase Micro Extraction-Gas Chromatography/Mass Spectrometry). It is widely used among researchers for milk and dairy products [11,12,13,14,15] and other foods [16,17,18]. It is used also in the flavoromics approach [19,20], because it is capable of ppb detection level for a wide range of molecular weight. Such a kind of studies is very useful for fresh foods, which are preferred by modern consumers and are highly perishable. For cheeses, too, the consumer preference is increasingly orienting towards the fresh types, whose production volume in EU in 2019 was about 3.5 million tons (about 34% of total cheese manufactured) [21]. Several strategies have been proposed for slowing down their decaying process, including high pressure and x-ray treatments, modified atmosphere and active packaging and use of antimicrobial compounds [22,23,24,25]. Stracciatella is an Italian traditional fresh cream cheese that is emerging on the international market. It is made up of mixture of double cream and thin Mozzarella strands (manually shredded) [26]; it is commonly sold packaged in polyethylene trays heat-sealed with laminated film. Due to high perishability, it must be kept refrigerated until consumption but, being the signs of alteration only recognizable after tasting, a non-negligible part of the product sold in large-scale retail stores turns into waste. High perishability of stracciatella is mostly caused by fast fat oxidation and microbial growth, favored by high pH and moisture content (6.0–6.2 and 62–65%, respectively) [27]. Efforts for preserving shelf life and sensory characteristics of this cheese have reached poor results. Only little shelf-life extension was obtained by Gammariello et al. [28], by applying modified atmosphere packaging (MAP), and by Conte et al. [29] combining it with two antimicrobial compounds (lysozyme and EDTA). Dambrosio et al. [30] and Rea et al. [31] concluded that the principal obstacle lay in the manufacturing process that involves intense manipulation. Unfortunately, the stracciatella production process cannot be mechanized since it is impossible to reproduce the results obtained by the manual procedure (in particular the mozzarella shredding phase). The only strategy that can be used to keep longer the original sensory characteristic is the addition of preservatives. Unfortunately, only a few molecules are allowed by EU legislation in fresh dairy products, and the most commonly used is sorbic acid. This preservative is able to inhibit the growth of numerous microorganisms, depending on the types, species and strains, but also on substrate properties and environmental factors. According to the Codex Alimentarius Commission, the maximum concentration in fresh cheese (as total sorbates) is 1000 mg kg^−1^. Even though sorbic acid has a GRAS (Generally Recognized As Safe) status, the dairy industries are asking for more natural preservatives. There is no information about recently studied aqueous extract obtained from olive leaf (OLE) in fresh cheeses. Its contribution on shelf life and chemical and sensory characteristics of stracciatella is still unknown. OLE has been recently tested for antimicrobial activity based on the high polyphenols content [32,33,34].

The aim of the present work was to study the relationships between the VOC profile, sensory features and microbiological characteristics of stracciatella cheese during storage, as affected by the addition of sorbic acid and OLE.

## 2. Materials and Methods

### 2.1. Preservatives Concentration and Sample Preparation

According to Ranieri et al. [35] OLE was produced at the laboratory scale, then its concentration to be added to cheese was chosen on the basis of both of the data reported in the literature and deriving from sensory preliminary trials. According to Caponio et al. [36] and Difonzo et al. [37], OLE added in an amount up to 1000 mg kg^−1^ was able to extend the shelf life of vegetable foods. The preliminary trials (data not shown) highlighted that OLE added at a concentration of 800 mg kg^−1^ negatively affected the sensory properties, causing an unpleasant odor and loss of freshness and frankness of flavor, while when present at a level of 400 mg kg^−1^ the typical aroma was maintained. A commercial food grade sorbic acid was supplied by Farmalabor (Canosa, Italy), and the level of addition was fixed at the maximum level (1000 mg kg^−1^) allowed by European legislation in fresh dairy products [38].

Four types of stracciatella samples were prepared: 3 experimental, containing different types of antimicrobials (OLE, sorbic acid and a mixture of the two), plus a control (Table 1). The antimicrobials were added to 30% fat UHT (Ultra High Temperature) cream, then the cream was mixed with freshly prepared mozzarella strands (1:1 *w/w*) and gently homogenized at room temperature in a kneader (40 rounds per minute applied for 5 min). The obtained samples were stored at 8 °C in plastic trays mechanically sealed with laminated film. OLE, obtained as reported in Difonzo et al. [39], had a total phenol content of 151 mg gallic acid equivalent (GAE) g^−1^ and a value of 950 µmol Trolox equivalent (TE) g^−1^. The samples were named Ex (sample with OLE), So (sample with sorbic acid), Ex-So (sample with OLE + sorbic acid) and Ctr (control). They were analyzed after 1 day and every 4 days from production until they resulted in being altered.

### 2.2. Chemical, Sensory and Microbiological Analyses

#### 2.2.1. Volatile Organic Compounds (VOCs)

VOCs were extracted at 37 °C for 15 min as reported in a previous paper [40] after addition of 3-pentanone (81.3 ng) as an internal standard for semi-quantitation. A Triplus RSH autosampler was used, equipped with a divinylbenzene/carboxen/polydimethylsiloxane 50/30 mm SPME fiber assembly (Supelco, Bellefonte, PA, USA). The fiber was desorbed at 220 °C for 2 min in the injection port of the gas chromatograph operating in the splitless mode. The GC–MS analysis was performed using a Trace 1300 chromatograph equipped with a capillary column VF-WAX MS (60 m, 0.25 mm) and connected to mass spectrometer ISQ Series 3.2 SP1 (Thermo Scientific, Waltham, MA, USA). The operating conditions were: oven temperatures, 50 °C for 0.1 min then 13 °C min^−1^ up to 180 °C and 18 °C min^−1^ up to 220 °C with an isothermal for 1.5 min. The mass detector was set at 1700 V voltage; source temperature, 250 °C; ionization energy 70 eV and scan range 33–200 amu. Peak identification was done by means of Xcalibur V2.0 Qual Browse software (Thermo Fisher Scientific, Waltham, MA, USA) by matching with the reference mass spectra of the NIST (National Institute of Standards and Technology, Gaithersburg, MD, USA) library.

#### 2.2.2. Antioxidant Activity and Oxidative Stability

The ABTS-TEAC (2,2′-azino-bis(3-ethylbenzothiazoline-6-sulfonic acid)/Trolox^®^-Equivalent Antioxidant Capacity) assay was carried out as described by Ranieri et al. [35] and the results were expressed as µmol TE g^−1^. Induction time was determined by RapidOxy (Anton Paar Prove Tec GmbH, Blankenfelde-Mahlow, Germany) measuring the time needed for a 10% drop of the oxygen pressure. The set parameters were the following: T = 140 °C, P = 700 kPa [38].

#### 2.2.3. Color

Colorimetric readings were carried out under D65 illuminant by using a spectro-colorimeter CM-700d (Konica Minolta Sensing, Osaka, Japan) equipped with a pulsed xenon lamp. The analysis was performed by placing the sample in a transparent quartz container. Lightness (L*), redness (a*, ±red-green) and yellowness (b*, ±yellow-blue) were determined coordinates in the CIE (Commission Internationale de l’Éclairage) color space [41].

#### 2.2.4. Organic Acids and pH

Organic acids were extracted as reported by Buffa et al. [42]. Separation was carried out on a Synergy Hydro RP column 80 Å, 4 µm, 250 mm × 4.6 mm (Phenomenex, Torrance, CA, USA), installed on a Waters HPLC composed of 600E pumps and a 996 diode array detector (Waters Corporation, Milford, CT, USA). Mobile phases were 0.1% orthophosphoric acid in water (eluent A) and acetonitrile (eluent B). The gradient was 0–18 min 100% A at 1 mL min^−1^ flow rate, then 18–18.3 min from 100% to 20% A; 18.3–19.5 min increasing flow rate to 1.4 mL min^−1^, then 19.5–22.5 isocratic and 22.5–23 min from 20% to 100% A and 23–43 min isocratic. Detection was done at *λ* = 214 nm. A pH meter equipped with a dairy specific electrode (FC2020, Hanna instruments, Woonsocket, RI, USA) was used for pH measurement.

#### 2.2.5. Sensory Analysis

Sensory analysis was assessed by a trained panel consisting of nine experts (4 women and 5 men, ranging in age from 25 to 60 years) selected according to the ISO 8586-1993 method. All of them were members of the Italian Association of Cheese Tasters (ONAF) and had attended a 20 h course about evaluation of cheese texture and flavor. Evaluation was carried by a quantitative descriptive analysis (QDA) and the samples were presented in a randomized and balanced way, in white disposable dishes coded by three-digit codes. The panelists described the cheese using sensory attributes chosen from the ONAF (Italian Organization of Cheese Tasters) vocabulary [43] and by scoring their intensity from 0 to 10. In the case of perception of an attribute not included in the vocabulary, it was only considered if the “weight percentage” (frequency of citations × perceived intensity) was more than 30%.

#### 2.2.6. Cultivable Microbiota

Microbiological analyses were performed according to methods described in Minervini et al. [44], using culture media and supplements purchased from Oxoid (Oxoid Limited, Basingstoke, UK), starting from 20 g of stracciatella. The microbial groups counted were: total mesophilic aerobic (plate count agar at 30 °C for 48 h), presumptive mesophilic lactobacilli (MRS agar with 0.1 g L^−1^ cycloheximide at 30 °C for 48 h), presumptive mesophilic cocci (M17 agar with 0.1 g L^−1^ cycloheximide at 30 °C for 48 h), enterococci (Slanetz and Bartley agar at 37 °C for 48 h), staphylococci (Baird Parker agar supplemented with egg yolk tellurite at 37 °C for 48 h), Enterobacteriaceae (VRBGA at 37 °C for 24 h), *Pseudomonas* spp. (*Pseudomonas* agar with CFC supplement at 30 °C for 24 h), yeasts (wort agar supplemented with 0.1 g L^−1^ chloramphenicol at 30 °C for 48 h) and molds (potato dextrose agar supplemented with 0.1 g L^−1^ chloramphenicol at 25 °C for 5 days). The microbiological counts were confirmed by taking representative colonies for each medium, and checking them for morphology, motility, Gram staining reaction and catalase test.

### 2.3. Statistical Analysis

The data were statistically processed by one-way ANOVA followed by Tukey’s HSD procedure at *p* < 0.05 using XLSTAT software (Addinsoft, NY, USA). Each sample was analyzed in triplicate. The data from the sensory analysis were also processed for generalized Procrustes analysis (GPA) with the same software.

## 3. Results and Discussion

### 3.1. VOC

**Figure 1:**
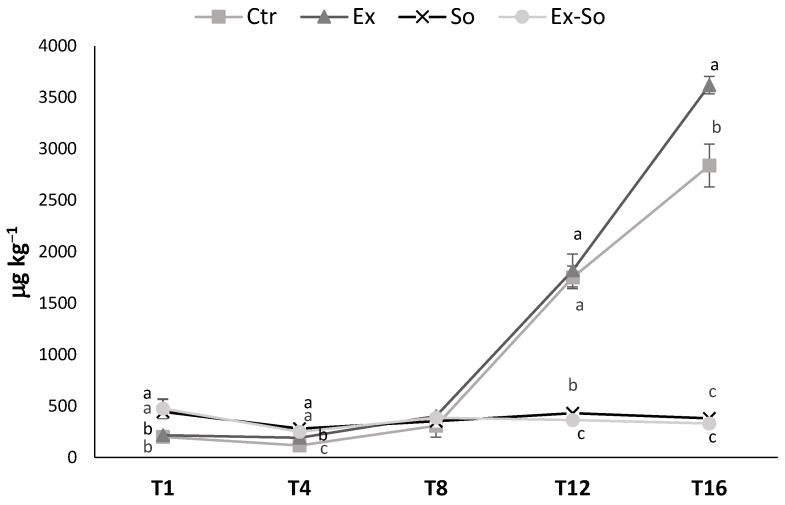
Total volatile compounds in stracciatella cheese during storage at 1 (T1), 4 (T4), 8 (T8), 12 (T12) and 16 (T16) days. Different letters above the lines indicate statistically different values (*p* < 0.05).

Figure 1 shows the total amounts of volatile compounds during storage of the samples. As expected, low quantities were detected in the early days, ranging from 200 to 470 μg kg^−1^. Until day 8, the differences among samples were not very relevant, even though they were sometimes statistically significant. Huge differences started after this time, with Ex and Ctr samples showing an increase to about 1800 μg kg^−1^, then to about 2700 and 3600 μg kg^−1^ (at day 16), respectively. Differently, the concentrations in Ex-So and So remained almost constant during time. Increase in VOC formation during storage was expected, considering the typical chemical and microbiological characteristics of the product [27,30,31], and from these quantitative data it clearly appeared that a significant inhibition of their formation was associated to the presence of sorbic acid. In order to better understand the mechanisms of formation, the entire VOC data set was grouped into seven chemical classes (Table 2). The samples were rather similar at day 1 as to the most abundant classes (ketones, esters and aliphatic hydrocarbons), whereas some differences were found in some less represented groups such as aldehydes, alcohols and sulfur compounds, whose content is known to be influenced by manipulation (they are highly produced by spoilage microorganism). The main difference at T1 accounted on acids, which were more abundant in So and Ex-So, but it was mainly due to the presence of sorbic acid added as a preservative. As already observed under the quantitative point of view, a clear qualitative differentiation among samples started from day 8, and the pair Ctr/Ex and Ex-So/So behaving quite similarly until the end of storage. At T8 aldehydes and alcohols resulted to be higher in the pair Ctr/Ex, whereas esters and acids were more abundant in Ex-So/So. The control sample had the highest concentration of sulphur compounds and the lowest of aliphatic hydrocarbons. The higher concentrations of aldehydes and alcohols in Ctrl and Ex could be the consequence of two different phenomena: (i) lipid oxidation, which is a common source of aldehydes formation in fresh cheeses, from whose dehydrogenation the corresponding alcohols can successively derive [3,45] and (ii) microbial metabolism. At the end of storage (T16) almost all chemical groups were much higher in Ctr and Ex, whereas only sulphur compounds were more abundant in Ex-So. Finally, very low concentration of aliphatic hydrocarbons characterized the Ex-So sample.

As to the single VOC, 41 molecules were identified in the entire set of samples, the most abundant compounds detected at the end of the storage period are reported in Table 3. Acetaldehyde and 3-methylbutanal were found only in Ex and Ctr. Acetaldehyde can be formed throughout different pathways: ethanol oxidation should be a highly probable pathway, considering that ethanol concentration was very high in these samples (713.6 and 556.4 μg kg^−1^ for Ex and Ctr respectively versus 7.8 and 4.2 μg kg^−1^ for Ex-So and So, respectively). The mechanism based on phenolic compounds oxidation could also have played a role in Ex, considering the presence of polyphenols from the leaf extract [3]. 3-methylbutanal in cheese often arise from the conversion of the corresponding amino acid (isoleucine) by the LAB activity [46], and its concentration suggests a more marked microbial activity with respect to the samples without sorbic acid. This hypothesis was supported by the huge increase of ketones in Ctr and Ex, mainly connected to the formation of acetoin (1839.57 and 1512.48 μg kg^−1^ respectively, versus 7.8 and 7.4 μg kg^−1^ in So and Ex-So) that commonly derives from LAB and yeasts metabolism [47]. Ethyl acetate and ethyl butanoate increased much more in Ex, in connection with a higher presence of ethanol, which represents the limiting factor for their formation [48]. This aspect was probably connected to the absence of sorbic acid acting against yeasts. On the other hand, the presence of this preservative in So and Ex-So gave rise to the formation of ethyl sorbate. Among alcohols, besides ethanol, also 3-methyl-1-butanol greatly characterized the samples without sorbic acid, suggesting a higher microbial activity in them. In some food matrices such as fruit juices, 3-methyl-1-butanol and phenylethyl alcohol have been related to yeast metabolism [3]. The former was reported by Morales et al. [49] as an important component of volatilome of Enterobacteriaceae strains of dairy origin. Other volatiles that could be connected to faster microbial growth in Ctr and Ex samples were sulphur compounds (i.e., dimethyl sulfide) that are formed, for instance, by some Enterobacteriaceae species [7], and organic acids such as acetic, butanoic and hexanoic.

### 3.2. Sensory Analysis and Possible Connections with VOC

**Figure 2:**
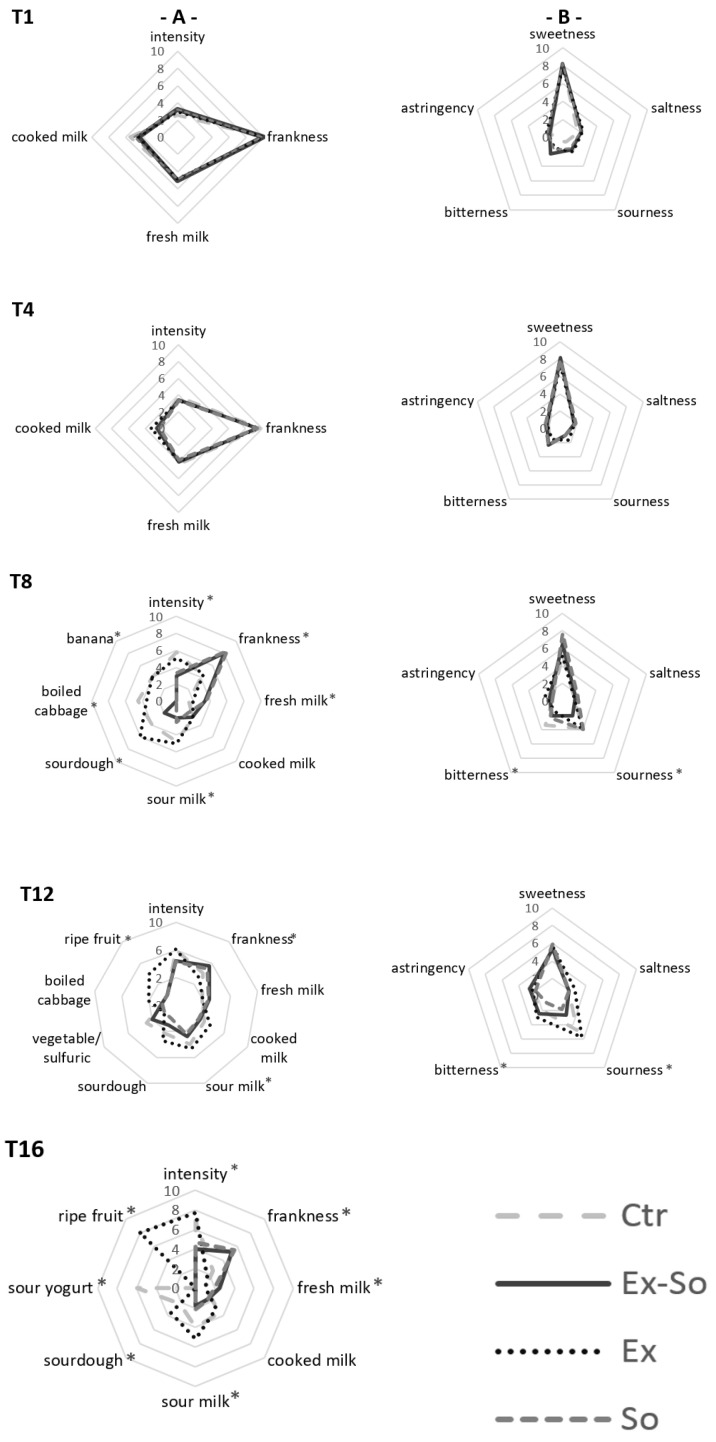
Quantitative descriptive analysis (QDA) of stracciatella odor (**A**) and taste perceptions (**B**). * = statistically different values (*p* < 0.05). Sampling time is the same as Figure 1.

**Figure 3:**
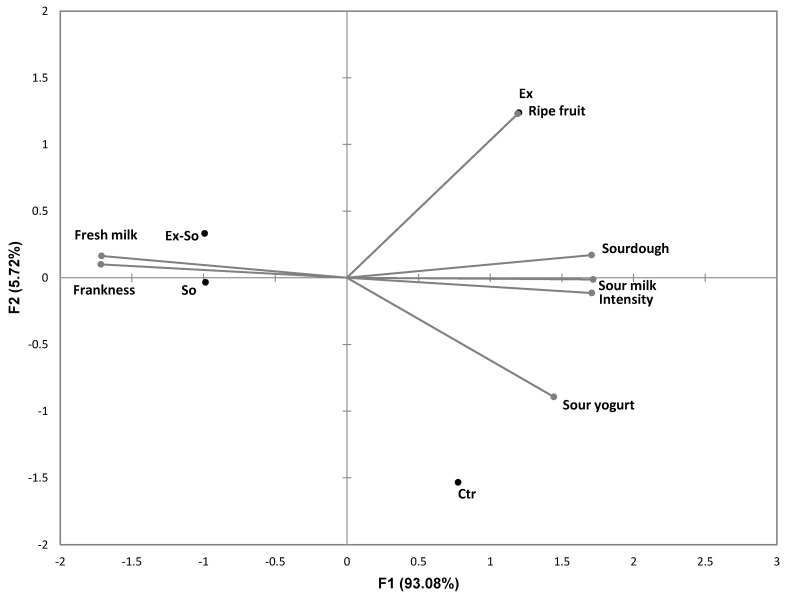
Generalized Procrustes analysis (GPA) of the dataset of the odor sensory analysis.

Figure 2 shows the quantitative descriptive analysis (QDA) graphs divided into odor (A) and taste (B) perceptions. All fresh samples had almost the same olfactory characteristics until day 4, demonstrating the absence of any influence of the preservatives on the sensory profile. Starting from day 8, in perfect agreement with the evolution of VOC, a loss of the freshness characteristics was observed in Ctr and Ex. In particular, the overall intensity increased, and considering that stracciatella must present a mild and delicate aroma, it has to be considered a symptom of ongoing changes. This variation led to the loss of “frankness” defined as the characteristic aroma of the product; with time, undesired notes of sour milk, sourdough, banana and boiled cabbage were also perceived. These results matched well with the increase of some VOC compounds, such as butanoic acid, ethyl esters, ethanol, dimethyl sulphide and 3-methylbutanal. The same trend was also observed for Ex-So and So, but only starting from day 12. At day 16 Ctr and Ex samples were totally unacceptable, differently from Ex-So and So. This finding could be related to the strong increase of some VOC that can be considered as responsible of off-flavors. As to taste (B) no differences were observed among samples until day 8, then Ctr and Ex started to lose sweetness and to evidence bitterness and sourness; a slight increase in sourness was also perceive in So. The responsibility of these changes could be attributed to the formation of free hydrophobic amino acids deriving from proteolysis and/or increase of organic acids. At day 12, Ex and Ctr were rejected for excessive sourness and sweetness decreased in Ex-So and So, but they were still judged as acceptable. At day 16 also these latter samples were rejected for bad smell (they were not tasted). Figure 3 shows the GPA plot based on the dataset of odor sensory analysis. Here it is possible to observe the distance among samples on a bidimensional plot. The two dimensions account for 98.80% of the total variance. Most of the differences (93.08%) can be explained on F1, where So and Ex-So were positioned on the left side of the plot, correlated to fresh milk and frankness perceptions. Ex and Ctr were on the right side of the plot, very far between them and related to ripe fruit and sour yogurt respectively, and in both cases also with sourdough, sour milk and odor intensity. This information allows us to make connections between the VOC profile and sensory analyses: So and Ex-So resulted to be quite similar, while Ex and Ctr were far from them and strongly affected by off-flavors. Thus, using sorbic acid or a mixture of the two preservatives seems to not interfere negatively on the product.

The potentially involved molecules in stracciatella flavor were hypothesized by calculating the odor active value (OAV) of VOC according to Qian et al. [50], on the basis of the odor thresholds in air taken from the literature. The VOC having OAV > 1 are shown in Table 4. Ethyl-acetate exceeded a value of 1 in all samples at any time, and should be one of the key odorants in this cheese. It presents an ethereal and green note and was found at the highest level in Ex, where also its precursors, ethanol and acetic acid, were at the highest level than all samples. Hexanal (green grass odor) should only play a role in fresh cheese, since OAV > 1 was only found at day 1. Surprisingly, ethanol was only present at day 1 with OAV = 1.3 in So and Ex-So, but the reason was unclear. As expected, it was not newly formed in these samples due to the inhibition of yeasts, whereas it strongly increased in Ctr and Ex, reaching a maximum OAV value of 69.6 and 89.2 at day 16 respectively. Other two potentially aroma-active esters, ethyl butanoate and ethyl hexanoate responsible of banana and apple notes [51], increased faster in Ctr and Ex. In these two samples, also acetoin (buttery and creamy odor) could contribute to the aroma, whereas in So and Ex-So it remained under the perception threshold. At day 8, also 3-methylbutanal (ethereal and fruity odor, but acrid when present at high concentration) exceeded a value of 1 in Ex and Ctr.

### 3.3. Chemical and Microbial Analyses

#### 3.3.1. Antioxidant Activity and Color

**Figure 4:**
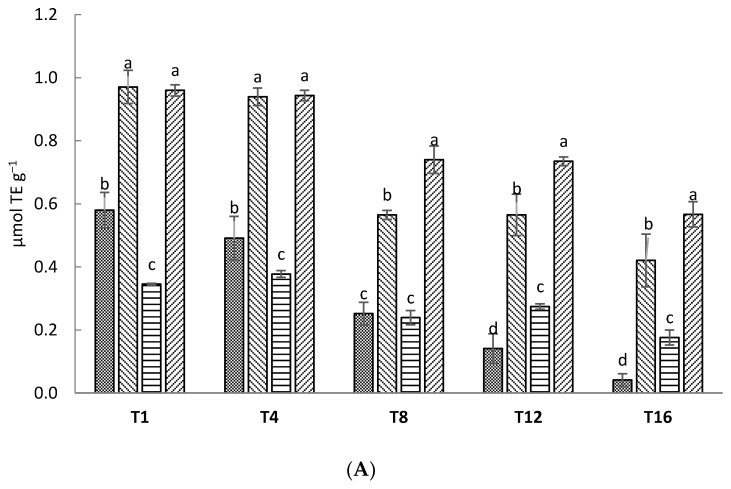

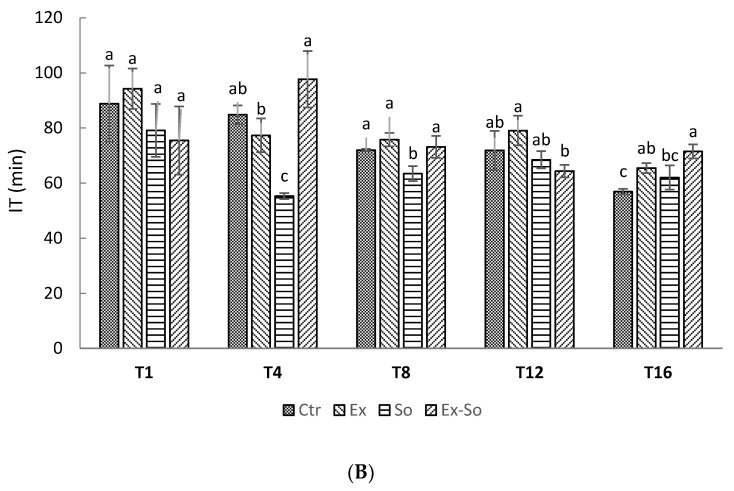
Antioxidant activity evaluation by means of ABTS-TEAC (2,2’-azino-bis(3-ethylbenzothiazoline-6-sulfonic acid)/Trolox^®^-Equivalent Antioxidant Capacity) (**A**) and oxidative stability measurement by RapidOxy (**B**). Sampling time is the same as Figure 1. Different letters at each sample indicate statistically different values (*p* < 0.05). Abbreviations: IT, Induction time.

As shown in Figure 4A, Ex and Ex-So samples had significantly higher antioxidant activity than Ctr and So throughout the entire storage period. Similar results were reported in studies on different food matrices [38,52]. The oxidative stability test carried out by RapidOxy gave different results (Figure 4B). No significant differences between samples were found at day 1, the So samples had the lowest induction time values after 4 and 8 days, and the Ex-So samples showed a higher value than Ctr at day 16, in accordance with antioxidant activity. These results could be due to the OLE effect, but the synergy with sorbic acid and interaction with the food matrix cannot be excluded. Overall, the results indicated that OLE, both added alone and together with sorbic acid, exerted an antioxidant activity in cheese. Different authors showed the antioxidant effect exerted by OLE in dairy products and according to literature the antioxidant power was lower in Ctr in a range of 40–80% and decreased during storage [53,54]. From this outcome it can be hypothesized that the higher presence of aldehydes in the VOC profile of OLE samples was mostly due to microbial activity rather than to fat oxidation.

As to color (Table 5), the values of some indices changed with time, but no significant differences were observed among samples taken at the same day. L* index decreased, in accordance with other authors [31,36], and according to García-Pérez et al. [55] it should be connected to the acidification of the product. Differently, the a* index slightly increased during storage, whereas b* index showed a non-defined trend. The increase of a* value could originate from polymerization of polyphenols and related tendency to browning [56].

#### 3.3.2. Organic Acids and pH

**Figure 5:**
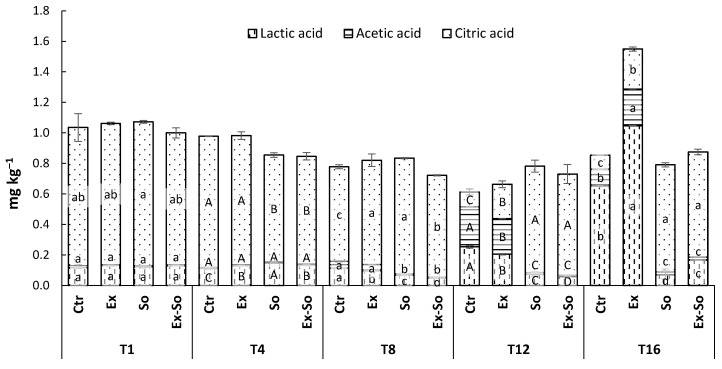
Trend of organic acids in stracciatella cheese during storage. Sampling time is the same as Figure 1. Different letters at each sample time for each acid indicate statistically different values (*p* < 0.05).

The changes in organic acids in dairy products are highly connected to microbial activities. In bottled milk and non-fermented products, as is stracciatella, they are undesired since they are responsible of off-flavors. From Figure 5, it can be observed that changes started early in Ctr and Ex samples: being a fermentable substrate, citric acid decreased from 0.91 and 0.93 mg kg^−1^ to 0.09 and 0.26 mg kg^−1^, respectively; lactic acid increased from 0.12 and 0.13 mg kg^−1^ to 0.65 and to 1.05; acetic acid increased from 0.01 to 0.11 and to 0.24 mg kg^−1^. There is no information concerning organic acids content of stracciatella cheese, but looking at other dairy products it is possible to confirm our results. In fact, citric acid is often involved in the Krebs cycle, and this justifies its reduction. Moreover, the increase in other organic acids (i.e., acetic and citric) is explained by higher microbial activities during storage [57,58,59]. According to Adda et al. [60] citric acid is used as a substrate by the microorganism to produce pyruvic and acetic acid, this finding clearly suits with our results. Differently, only slight variations were observed in the other samples containing sorbic acid or a mixture of the two preservatives. These results matched well with the decreasing trend of pH that went from 6.34 to 5.89 in Ctr and from 6.29 to 5.83 in Ex; in contrast, pH remained almost unchanged in So and Ex-So. The lower values in these latter samples found already at day 1 (6.02 and 6.14, respectively) were caused by the acidic properties of sorbic acid (pKa 4.76). These findings highlight a possible antimicrobial effect of olive leaf extract in synergy with sorbic acid.

#### 3.3.3. Cultivable Microbiota

**Figure 6:**
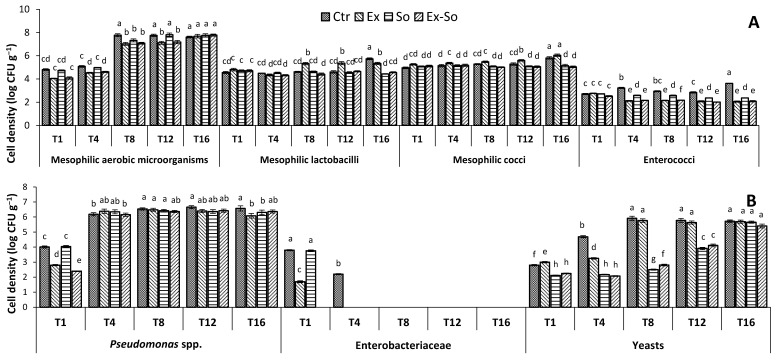
Cell numbers (log CFU g^−1^, average values of 3 triplicates ± SD) of mesophilic aerobic microorganisms, presumptive mesophilic lactobacilli, mesophilic cocci and enterococci (**A**), *Pseudomonas* spp., Enterobacteriaceae and yeasts (**B**) in stracciatella samples. Ctr = control; Ex = added with olive leaf extract; So = added with sorbic acid; Ex-So = added with both sorbic acid and olive leaf extract. Sampling time is the same as Figure 1. Different letters for each microbial group indicate significant differences at *p* < 0.05.

The evolution of cultivable microbiota during cheese storage matched well with the results of the chemical and sensory analyses. It changed with different magnitudes in samples during storage, depending on the storage time and microbial group (Figure 6). The cell densities of mesophilic aerobic microorganisms ranged from about 4.1 (Ex and Ex-So) to 4.81 (Ctr) log CFU g^−1^ after 1 day of storage. At the end of shelf life, the values were higher than 7.0 log CFU g^−1^ in all samples, without significant differences (*p* > 0.05) between experimental and control. However, preservatives exerted an effect during storage, since the highest cell load was reached more rapidly (at 8 days) in Ctr than in Ex and Ex-So samples (at 16 days). Thus, the probability to find earlier VOC responsible for off-flavor in these samples was higher. Lactic acid bacteria (LAB) in stracciatella are considered altering agents, just like in bottled milk. The average cell densities of mesophilic lactobacilli and cocci at day 1 were 4.7 and 5.1 log CFU g^−1^, respectively, while enterococci were about 2.6 log CFU g^−1^, without significant differences among samples (*p* > 0.05). Lactobacilli and cocci remained almost constant during storage in So or in Ex-So samples, whereas their number increased up to 1 log cycle in Ctr and Ex cheeses at 16 days. Additionally, enterococci increased up to 1 log cycle at the end of storage, but only in Ctr (about 3.6 log CFU g^−1^), whereas it decreased in all other samples. These findings suggest that OLE was effective in controlling enterococci when used alone, whereas it only had an effect on the other two LAB groups when associated to sorbic acid. Our results corroborate the findings of Roila et al. [61] and Servili et al. [62], who verified that the inhibitory effects of a polyphenol extract from an olive oil byproduct against lactic acid bacteria in mozzarella and a fermented milk beverage were scarce and dose-dependent. According to Hurtado et al. [63], some LAB strains can degrade oleuropein and metabolize specific compounds of OLE. In addition, the antimicrobial activity of the leaf extract in dairy products may be partially inhibited by the chemical interaction between phenolic hydroxyl groups and milk proteins [64]. *Pseudomonas* spp. are the most feared spoilage bacteria in fresh cheeses, since they can replicate at refrigeration conditions [65,66]. After 1 day of storage, the two cheeses containing OLE showed a lower (*p* < 0.05) cell density of *Pseudomonas* spp. than control. Successively, the counts increased in all samples reaching the maximum cell densities of about 6.3 log CFU g^−1^. Despite the effect observed at the early stage, OLE did not work against these bacteria, both alone and in the presence of sorbic acid. Inefficacy could be due to two main reasons: (1) OLE concentration was too low to have an effect. In fact, antimicrobial activity of olive polyphenols against Gram-negative bacteria, such as *Escherichia coli* and *Pseudomonas*, was reported to be dose-dependent [61,67] and (2) pH of the cheese (6.0–6.2) was too high for allowing efficacy of sorbic acid: in cottage cheese it acted against *Pseudomonas* at pH 4.6–5.1 [68]. As for Enterobacteriaceae, after the first day of storage significant differences were observed between Ctr (3.8 log CFU g^−1^) and So (3.7 log CFU g^−1^) compared to Ex (1.7 log CFU g^−1^) and Ex-So (<1 log CFU g^−1^) samples. Successively, they disappeared more rapidly in the cheeses added with antimicrobials. This finding is of particular relevance, because Enterobacteriaceae are involved in spoilage phenomena in the dairy product. In accordance with VOC results, Enterobacteriaceae are known as producers of some VOC as 3-methyl-1-butanol, found only in samples without sorbic acid [49,69]. Staphylococci and molds were not detected, differently from yeasts. Overall, effectiveness of OLE in controlling yeasts was ascertained at early phases (day 4), successively it had an effect only in the presence of sorbic acid, confirming the usefulness of sorbic acid in controlling mold and yeast.

## 4. Conclusions

The results of the present work pointed out the importance of relating the VOC profile and sensory results as a decay’s symptom in fresh cheeses. In particular, VOC analysis could be a fundamental tool for predicting the shelf life of stracciatella, together with the classic microbiological and sensory assays. In addition, it could help to monitor the formation of some key volatiles responsible for an off-flavor and prevent rejection of the product by consumers in large-scale retails. The study allowed us to suitably evaluate the influence of the preservatives on the sensory characteristics. The samples added with sorbic acid or a combination of sorbic acid and olive leaf extract gave the best result as to the evolution of the VOC profile. It was reflected in better flavor preservation during storage, allowing us to better preserve both stracciatella’s delicate aroma and taste. The color parameter results did not show any relevant difference among products taken at the same day, so the addition of preservatives does not influence the product aspect. Although stracciatella OLE-enriched samples reached higher antioxidant activity than Ctr and So, such performances were mostly connected to the well-known antimicrobial effect of sorbic acid, also further to a possible synergic effect with OLE, in particular against *Enterococcus* spp. and Enterobacteriaceae. Possibly better results might be reached also against other spoilage microorganisms by increasing the extract concentration, but the negative impact on the sensory characteristics appears to be a main limit. Nevertheless, the possible synergic effect could help producers to reduce the concentration of chemical preservatives. A further synergic effect could be obtained by slight lowering of pH of the cheese, compatibly with maintaining the flavor acceptable.

## Figures and Tables

**Table 1:** Ingredients and preservatives concentration used to produce stracciatella cheese.

	CTR	Ex	So	Ex-So
Cream/mozzarella strands (*w/w* as *g*)	500/500	500/500	500/500	500/500
OLE (mg kg^−1^)	-	400	-	400
Sorbic acid (mg kg^−1^)	-	-	1000	1000

**Table 2:** Evolution of volatile compounds (μg kg^−1^; mean values and standard deviation) during stracciatella cheese shelf life.

Parameter	T1				T4				T8				T12				T16			
	Ctr	Ex	So	Ex-So	Ctr	Ex	So	Ex-So	Ctr	Ex	So	Ex-So	Ctr	Ex	So	Ex-So	Ctr	Ex	So	Ex-So
Aldehydes	9.65 ^a^ ± 1.55	8.00 ^a^ ± 0.12	10.02 ^a^ ± 1.99	0.78 ^b^ ± 0.06	5.01 ^b^ ± 2.42	5.19 ^b^ ± 0.34	12.03 ^a^ ± 2.15	5.82 ^b^ ± 1.26	46.09 ^a^ ± 20.77	39.94 ^a^ ± 4.84	0.46 ^b^ ± 0.02	0.52 ^b^ ± 0.08	9.46 ^b^ ± 1.05	36.58 ^a^ ± 1.43	0.88 ^c^ ± 0.25	1.03 ^c^ ± 0.17	4.98 ^b^ ± 0.54	20.89 ^a^ ± 2.74	0.72 ^c^ ± 0.3	0.83 ^c^ ± 0.11
Ketones	57.04 ^a^ ± 11.9	62.83 ^a^ ± 0.92	67.84 ^a^ ± 11.34	58.57 ^a^ ± 4.4	58.36 ^b^ ± 10.68	78.65 ^ab^ ± 3.5	65.94 ^ab^ ± 10.59	81.26 ^a^ ± 2.19	105.58 ^a^ ± 32.84	96.70 ^a^ ± 1.86	80.20 ^a^ ± 15.25	93.16 ^a^ ± 2.97	1251.03 ^a^ ± 64.69	865.58 ^b^ ± 93.71	83.64 ^c^ ± 7.9	85.64 ^c^ ± 1.06	1894.70 ^a^ ± 111.37	1603.86 ^b^ ± 10.74	105.02 ^c^ ± 7.19	82.11 ^d^ ± 6.34
Esters	46.28 ^a^ ± 18.4	62.21 ^a^ ± 0.6	64.75 ^a^ ± 18.6	61.29 ^a^ ± 5.76	24.32 ^b^ ± 7.23	58.56 ^a^ ± 0.05	52.25 ^a^ ± 7.23	57.46 ^a^ ± 5.88	47.88 ^c^ ± 11.75	77.24 ^b^ ± 2.28	83.05 ^a^ ± 1.55	86.36 ^a^ ± 2.89	41.33d ± 3.68	178.18 ^a^ ± 4.07	80.11 ^b^ ± 0.06	75.04 ^c^ ± 0.99	134.08 ^b^ ± 7.24	187.41 ^a^ ± 7.08	77.62 ^c^ ± 14.99	103.54 ^c^ ± 11.02
Alcohols	22.16 ^ab^ ± 7.89	18.82 ^b^ ± 4.0	27.75 ^a^ ± 3.74	23.58 ^ab^ ± 1.98	27.00 ^a^ ± 2.92	13.68 ^c^ ± 0.42	17.55 ^b^ ± 1.53	17.36 ^b^ ± 1.14	76.38 ^a^ ± 25.79	115.65 ^a^ ± 15.62	18.08 ^b^ ± 0.52	18.16 ^b^ ± 2.96	372.65 ^b^ ± 21.01	629.53 ^a^ ± 32.08	20.50 ^c^ ± 5.29	30.49 ^c^ ± 5.51	659.30 ^b^ ± 60.15	1685.94 ^a^ ± 76.51	17.51 ^d^ ± 1.70	39.64 ^c^ ± 8.6
Sulfur compounds	0.43 ^ab^ ± 0.09	0.19 ^c^ ± 0.14	0.52 ^a^ ± 0.08	0.28 ^bc^ ± 0.06	0.31 ^b^ ± 0.03	0.41 ^a^ ± 0.04	0.40 ^ab^ ± 0.10	0.35 ^ab^ ± 0.04	4.88 ^a^ ± 1.13	0.51 ^b^ ± 0.09	0.58 ^b^ ± 0.02	0.64 ^b^ ± 0.04	11.35 ^a^ ± 3.38	0.54 ^b^ ± 0.12	0.73 ^b^ ± 0.07	0.80 ^b^ ± 0.19	3.82 ^b^ ± 0.03	3.90 ^b^ ± 0.62	2.18 ^c^ ± 0.15	7.51 ^a^ ± 0.75
Acids	5.53 ^b^ ± 1.89	3.29 ^b^ ± 0.35	212.66 ^a^ ± 33.12	275.92 ^a^ ± 35.46	2.66 ^c^ ± 0.76	4.42 ^b^ ± 0.21	89.75 ^a^ ± 16.42	85.54 ^a^ ± 7.65	26.68 ^b^ ± 19.02	17.95 ^b^ ± 2.74	89.99 ^a^ ± 8.93	114.94 ^a^ ± 33.11	47.55 ^c^ ± 10.53	80.32 ^bc^ ± 26.1	164.21 ^a^ ± 22.03	103.14 ^b^ ± 4.07	126.86 ^a^ ± 30.43	93.17 ^a^ ± 6.01	139.67 ^a^ ± 78.84	95.47 ^a^ ± 8.14
Aliphatic hydrocarbons	59.35 ^a^ ± 5.63	62.64 ^a^ ± 4.3	61.53 ^a^ ± 22.44	54.06 ^a^ ± 5.32	0.00 ^c^ ± 0.00	33.00 ^a^ ± 0.63	43.37 ^a^ ± 10.75	2.62 ^b^ ± 0.17	1.35 ^b^ ± 0.53	54.31 ^a^ ± 15.58	82.17 ^a^ ± 15.05	73.20 ^a^ ± 10.46	17.59 ^b^ ± 6.69	27.31 ^b^ ± 4.39	80.26 ^a^ ± 6.97	67.47 ^a^ ± 7.15	14.20 ^a^ ± 7.05	24.16 ^a^ ± 6.65	33.80 ^a^ ± 12.32	2.37 ^b^ ± 0.03

T1, T4, T8, T12 and T16, stracciatella analyzed after 1, 4, 8, 12 and 16 days, respectively. Ctr, stracciatella control; Ex, stracciatella with olive leaf extract; So, stracciatella with sorbic acid; Ex-So, stracciatella with olive leaf extract and sorbic acid. Values in the rows with different superscripts at each sampling time differ at *p* < 0.05.

**Table 3:** Main volatile compounds (μg kg^−1^) found at the end of shelf life (16 days) in stracciatella cheese (only compounds exceeding 1 μg are reported).

	Ctr	Ex	So	Ex-So	R
Aldehydes					
Acetaldehyde	3.7 ^b^ ± 0.5	12.0 ^a^ ± 2.3	-	-	Ms,Std
3-methylbutanal	0.9 ^b^ ± 0.1	8.4 ^a^ ± 0.3	-	-	Ms,Std
Ketones					
acetone	4.5 ^b^ ± 0.3	5.1 ^b^ ± 0.2	21.6 ^a^ ± 5.0	15.5 ^a^ ± 1.0	Ms,Std
2-butanone	15.9 ^b^ ± 0.9	55.0 ^a^ ± 2.4	19.4 ^b^ ± 2.8	19.0 ^b^ ± 0.8	Ms,Std
2-heptanone	19.7 ^c^ ± 0.6	22.4 ^c^ ± 0.8	49.9 ^a^ ± 4.1	32.9 ^b^ ± 2.5	Ms,Std
2-hydroxy-3-pentanone	11.8 ^a^ ± 1.6	5.1 ^b^ ± 0.6	-	-	Ms,Std
2-nonanone	2.4 ^b^ ± 0.2	2.8 ^b^ ± 0.2	5.0 ^ab^ ± 0.2	5.9 ^a^ ± 1.7	Ms,Std
acetoin	1839.5 ^a^ ± 109.0	1512.5 ^b^ ± 10.0	7.8 ^c^ ± 2.9	7.4 ^c^ ± 1.8	Ms,Std
Esters					
ethyl acetate	37.5 ^c^ ± 0.8	107.5 ^a^ ± 5.8	68.3 ^b^ ± 13.8	81.8 ^ab^ ± 0.3	Ms,Std
butanoic acid, ethyl ester	93.5 ^a^ ± 7.7	75.0 ^b^ ± 1.3	2.8 ^c^ ± 0.6	13.0 ^c^ ± 1.3	Ms,Std
2-butenoic acid, ethyl ester (E)	0.5 ^b^ ± 0.1	1.4 ^a^ ± 0.1	0.4 ^b^ ± 0.0	-	Ms
hexanoic acid, ethyl ester	2.0 ^a^ ± 0.3	3.2 ^a^ ± 0.1	0.1 ^b^ ± 0.0	2.2 ^a^ ± 0.8	Ms,Std
sorbic acid, ethyl ester	-	-	6.0 ^a^ ± 0.5	6.5 ^a^ ± 1.9	Ms
Alcohols					
ethanol	556.4 ^b^ ± 48.0	713.6 ^a^ ± 49.5	4.2 ^c^ ± 0.4	7.8 ^c^ ± 0.1	Ms,Std
1-propanol, 2 methyl-	9.7 ^b^ ± 0.6	76.3 ^a^ ± 1.7	3.2 ^c^ ± 0.3	-	Ms
1-butanol, 3-methyl	89.1 ^b^ ± 11.2	888.3 ^a^ ± 25.2	5.0 ^c^ ± 0.3	7.9 ^c^ ± 1.3	Ms,Std
1-pentanol	0.5 ^b^ ± 0.1	0.8 ^b^ ± 0.1	1.2 ^b^ ± 0.4	3.9 ^a^ ± 0.1	Ms,Std
4-methyl-2-hexanol	2.9 ^b^ ± 0.1	3.2 ^b^ ± 0.0	2.4 ^b^ ± 1.2	18.3 ^a^ ± 7.1	Ms
1-hexanol, 2-ethyl	0.4 ^b^ ± 0.1	0.9 ^ab^ ± 0.0	1.4 ^a^ ± 0.3	1.6 ^a^ ± 0.3	Ms
phenilethyl alcohol	0.1 ^b^ ± 0.0	2.7 ^a^ ± 0.1	0.1 ^b^ ± 0.0	0.1 ^b^ ± 0.0	Ms,Std
Sulfur Compounds					
dimethyl sulfide	3.5 ^b^ ± 0.0	3.6 ^b^ ± 0.5	1.4 ^c^ ± 0.2	6.8 ^a^ ± 0.7	Ms,Std
Acids					
acetic acid	19.6 ^b^ ± 8.4	52.2 ^a^ ± 5.4	3.4 ^c^ ± 0.2	10.1 ^b^ ± 3.1	Ms,Std
butanoic acid	43.9 ^a^ ± 12.2	14.6 ^b^ ± 0.3	5.8 ^c^ ± 3.4	5.9 ^c^ ± 1.5	Ms,Std
hexanoic acid	51.3 ^a^ ± 8.0	18.0 ^b^ ± 0.2	10.8 ^c^ ± 6.0	9.2 ^c^ ± 3.3	Ms,Std
octanoic acid	9.7 ^a^ ± 1.6	4.4 ^b^ ± 0.2	3.3 ^b^ ± 1.4	3.8 ^b^ ± 0.9	Ms,Std
propanoic acid, 2 methyl	0.1 ^b^ ± 0.0	1.4 ^a^ ± 0.0	-	-	Ms,Std
butanoic acid, 3-methyl	0.6 ^b^ ± 0.1	1.5 ^a^ ± 0.2	0.2 ^bc^ ± 0.0	-	Ms,Std
sorbic acid	-	-	115.9 ^a^ ± 68.3	66.3 ^a^ ± 0.7	Ms
Aliphatic hydrocarbons					
1-heptene, 2,4-dimethyl-	14.2 ^ab^ ± 2.0	24.1 ^ab^ ± 6.7	38.8 ^a^ ± 12.3	2.3 ^c^ ± 0.1	Ms

Ctr, stracciatella control; Ex, stracciatella with olive leaf extract; So, stracciatella with sorbic acid; Ex-So, stracciatella with olive leaf extract and sorbic acid. R = identification method; Ms = mass spectrometer; Std = chemical standard. Values in the rows with different letters differ at *p* < 0.05.

**Table 4:** Volatile compounds with odor active value (OAV) > 1 during stracciatella cheese shelf life.

Volatile Compound	Ctr					Ex					So					Ex-So					Odor Description
	T1	T4	T8	T12	T16	T1	T4	T8	T12	T16	T1	T4	T8	T12	T16	T1	T4	T8	T12	T16	
3-Methylbutanal	-	4.5	228.2	14.2	4.5	-	-	197.2	180.5	42.3	-	-	-	-	-	-	-	-	-	-	Powerful penetrating, fusty, acrid, apple-like
Hexanal	2.0	-	-	-	-	1.6	1.0	-	-	-	2.0	2.5	-	-	-	-	1.2	-	-	-	Green, fatty, fruity
Nonanal	-	-	-	-	-	-	-	-	-	-	-	-	-	-	-	-	-	-	1.0	-	Sweet, orange, orange peel
2-Nonanone	-	-	1.2	-	-	-	-	-	1.1	-	-	-	-	-	1.0	-	-	-	-	1.2	Fresh sweet, weedy, earthy, herbal
Acetoin	-	-	-	1.5	2.3	-	-	-	1.0	1.9	-	-	-	-	-	-	-	-	-	-	Intense buttery, creamy
Ethyl acetate	9.3	4.9	8.3	5.1	7.5	12.4	11.7	12.0	27.1	21.5	12.1	10.1	15.2	14.3	13.7	11.5	10.8	15.5	13.2	16.4	Ethereal, fruity, green
Butanoic acid, ethyl ester	-	-	45.4	149.8	935.0	-	-	131.2	302.1	749.8	-	-	28.7	31.9	27.9	-	-	45.2	38.9	130.1	Pineapple
Hexanoic acid, ethyl ester	-	-	3.8	1.3	6.8	-	-	7.6	36.6	10.8	-	-	1.3	1.4	-	-	-	2.1	5.5	7.2	Floral, fruity, apple, banana, pineapple
Ethanol	-	1.0	2.4	35.1	69.6	-	-	5.6	29.5	89.2	1.3	-	-	-	-	1.3	-	-	-	1.0	Pleasant, weak, ethereal, vinous
1-Butanol, 3-methyl	-	-	-	1.2	1.3	-	-	-	5.3	12.5	-	-	-	-	-	-	-	-	-	-	Banana, alcohol, fruity
Phenilethyl alcohol	1.4	-	-	-	-	-	-	-	-	2.3	1.0	-	-	-	-	-	-	-	-	-	Characteristic rose-like
Dimethyl sulfide	-	-	14.3	36.6	11.8	-	-	-	-	11.9	-	-	-	-	4.7	-	-	-	-	22.7	Unpleasant odor of wild radish, cabbage-like

Ctr, stracciatella control; Ex, stracciatella with olive leaf extract; So, stracciatella with sorbic acid; Ex-So, stracciatella with olive leaf extract and sorbic acid. T1, T4, T8, T12 and T16, stracciatella analyzed after 1, 4, 8, 12 and 16 days, respectively.

**Table 5:** Color parameters (mean values and standard deviation) during stracciatella cheese shelf life.

Parameter	T1				T4				T8				T12				T16			
	Ctr	Ex	So	Ex-So	Ctr	Ex	So	Ex-So	Ctr	Ex	So	Ex-So	Ctr	Ex	So	Ex-So	Ctr	Ex	So	Ex-So
L*	86.54 ^a^ ± 11.84	92.34 ^a^ ± 7.21	93.29 ^a^ ± 2.00	95.35 ^a^ ± 4.89	95.27 ^a^ ± 3.87	94.12 ^a^ ± 4.95	95.28 ^a^ ± 3.00	97.9 ^a^ ± 1.28	95.3 ^a^ ± 1.67	95.16 ^a^ ± 1.12	98.32 ^a^ ± 1.32	93.84 ^a^ ± 1.98	93.28 ^a^ ± 0.15	90.64 ^a^ ± 0.86	93.58 ^a^ ± 2.77	92.96 ^a^ ± 3.87	90.17 ^a^ ± 4.41	88.70 ^a^ ± 4.91	90.77 ^a^ ± 1.31	77.27 ^b^ ± 2.55
a*	−0.21 ^a^ ± 0.13	−0.38 ^a^ ± 0.12	−0.16 ^a^ ± 0.03	−0.3 ^a^ ± 0.03	−0.08 ^a^ ± 0.17	−0.23 ^a^ ± 0.10	−0.14 ^a^ ± 0.01	−0.38 ^a^ ± 0.08	−0.06 ^a^ ± 0.20	−0.28 ^a^ ± 0.12	−0.12 ^a^ ± 0.01	−0.25 ^a^ ± 0.18	0.10 ^a^ ± 0.05	0.06 ^a^ ± 0.09	0.02 ^a^ ± 0.04	−0.05 ^a^ ± 0.10	0.21 ^a^ ± 0.05	0.10 ^b^ ± 0.10	0.02 ^b^ ± 0.01	0.26 ^a^ ± 0.20
b*	8.70 ^a^ ± 0.49	8.67 ^a^ ± 0.10	8.36 ^a^ ± 0.21	8.93 ^a^ ± 0.57	7.52 ^a^ ± 0.12	8.51 ^a^ ± 0.39	9.08 ^a^ ± 0.91	8.16 ^a^ ± 0.69	8.84 ^a^ ± 0.16	10.0 ^a^ ± 0.17	8.31 ^b^ ± 0.46	8.79 ^b^ ± 0.21	8.79 ^b^ ± 0.06	9.97 ^a^ ± 0.43	9.08 ^a^ ± 0.99	9.28 ^a^ ± 0.52	8.67 ^a^ ± 0.10	9.17 ^a^ ± 0.50	9.17 ^a^ ± 0.25	7.61 ^b^ ± 0.76

T1, T4, T8, T12 and T16, stracciatella analyzed after 1, 4, 8, 12 and 16 days, respectively. Ctr, stracciatella control; Ex, stracciatella with olive leaf extract; So, stracciatella with sorbic acid; Ex-So, stracciatella with olive leaf extract and sorbic acid. Values in the rows with different superscripts at each sampling time differ at *p* < 0.05.
